# Towards a conceptual framework for explaining variation in nocturnal departure time of songbird migrants

**DOI:** 10.1186/s40462-016-0089-2

**Published:** 2016-10-17

**Authors:** Florian Müller, Philip D. Taylor, Sissel Sjöberg, Rachel Muheim, Arseny Tsvey, Stuart A. Mackenzie, Heiko Schmaljohann

**Affiliations:** 1 Institute of Avian Research “Vogelwarte Helgoland”, An der Vogelwarte 21, 26386 Wilhelmshaven, Germany; 2 Department of Biology, Acadia University, 33 Westwood Avenue, Wolfville, NS B4P 2R6 Canada; 3 Bird Studies Canada, 115 Front Street, Port Rowan, ON N0E 1M0 Canada; 4 Department of Biology, Lund University, Biology Building, Sölvegatan 35, 223 62 Lund, Sweden; 5 Biological Station Rybachy, Zoological Institute RAS, RU-238535 Rybachy, Kaliningrad region Russia

**Keywords:** Circannual and circadian rhythms, Departure, Innate migration program, Migration, Night, Songbird, Time

## Abstract

Most songbird migrants travel between their breeding areas and wintering grounds by a series of nocturnal flights. The exact nocturnal departure time for these flights varies considerably between individuals even of the same species. Although the basic circannual and circadian rhythms of songbirds, their adaptation to migration, and the factors influencing the birds’ day-to-day departure decision are reasonably well studied, we do not understand how birds time their departures within the night. These decisions are crucial, because the nocturnal departure time defines the potential flight duration of the migratory night. The distances covered during the nocturnal migratory flights in the course of migration in turn directly affect the overall speed of migration. To understand the factors influencing the arrival of the birds in the breeding/wintering areas, we need to investigate the mechanisms that control nocturnal departure time. Here, we provide the first conceptual framework for explaining the variation commonly observed in this migratory trait. The basic schedule of nocturnal departure is likely regulated by both the circannual and circadian rhythms of the innate migration program. We postulate that the endogenously controlled schedule of nocturnal departures is modified by intrinsic and extrinsic factors. So far there is only correlative evidence that birds with a high fuel load or a considerable increase in fuel load and significant wind (flow) assistance towards their migratory goal depart early within the night. In contrast, birds migrating with little fuel and under unfavorable wind conditions show high variation in their nocturnal departure time. The latter may contain an unknown proportion of nocturnal movements not directly related to migratory flights. Excluding such movements is crucial to clearly identify the main drivers of the variation in nocturnal departure time. In general we assume that the observed variation in the nocturnal departure time is explained by individually different reactions norms of the innate migration program to both intrinsic and extrinsic factors.

## Background

Each year billions of birds migrate between their breeding areas and wintering grounds [1, 2]. The vast majority of these are songbird migrants [1, 3] that follow a stop-and-go migration strategy [4, 5] with replenishing the fuel used during previous flights when stopping over [5, 6]. Most songbird migrants travel exclusively during the night [4, 7–9], likely because energetic costs [10], water loss [11, 12], and predation risk [13] are reduced in comparison to daytime travels [4, 14].

Migratory movements of nocturnal migrants are usually terminated before sunrise [4, 7–9], with only some exceptions when crossing ecological barriers [15–20]. Provided that these birds usually restrict their migratory activity to the night hours, their departure time within the night defines the potential flight duration, i.e., the potential time spent flying during a migratory flight bout, assuming a termination of flight shortly before sunrise. The flight duration and a bird’s ground speed represent the two core factors determining a bird’s travel speed [21, 22]. Travel speed and total stopover duration together define a bird’s total speed of migration. Variation in total speed of migration in turn affects the timing of the arrival at both the breeding areas and the wintering grounds, which has an effect on reproductive success and survival probability [23–26]. Understanding the causes of variation in nocturnal departure time and in nocturnal landing time is therefore important for explaining variation both in total speed of migration and the phenology of birds. In this review we focus on variation in nocturnal departure time, because there is little information available about when songbirds terminate their nocturnal migratory flights.

**Fig. 1:**
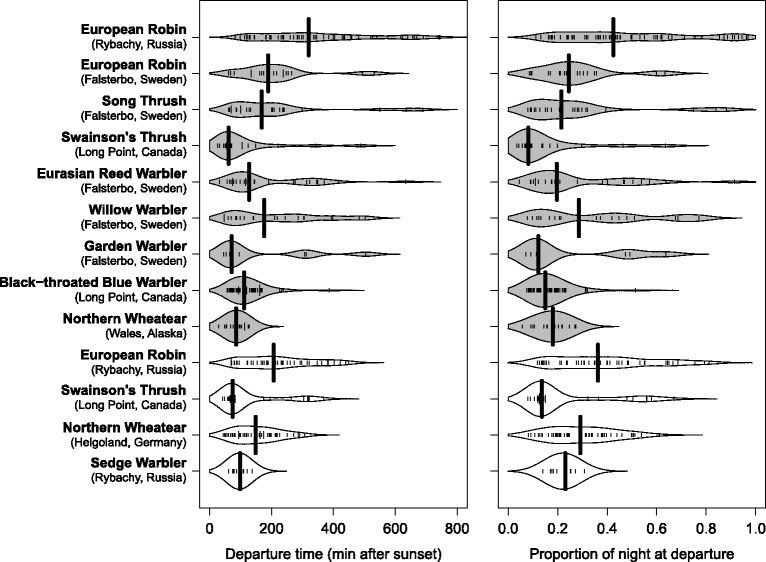
Variation in the nocturnal departure time of different songbird species as obtained from different radio tracking studies. Bean plots illustrate variation in nocturnal departure timing in relation to sunset (*left side*) and the proportion of the night (*right side*). Extension of the bean plots represent kernel density estimations of the distribution of individual departures, given as small lines in the plot. Broad lines represent the median nocturnal departure time of the respective group. *Gray bean plots*: autumn migration; *white bean plots*: spring migration. European Robin (*Erithacus rubecula*) at Rybachy, Russia [39] and Falsterbo, Sweden [46]; Song Thrush (*Turdus philomelos*) at Falsterbo, Sweden [46]; Swainson’s Thrush (*Catharus ustulatus*) at Long Point, Ontario, Canada [49, 50]; Eurasian Reed Warbler (*Acrocephalus scirpaceus*) at Falsterbo, Sweden [37]; Willow Warbler (*Phylloscopus trochilus*) at Falsterbo, Sweden [46]; Garden Warbler (*Sylvia borin*) at Falsterbo, Sweden [46]; Black-throated Blue Warbler (*Setophaga caerulescens*) at Long Point, Ontario, Canada [49, 50]; Northern Wheatears (*Oenanthe oenanthe*) at Wales, Alaska, USA [44] and on Helgoland, Germany [42, 43]; Sedge Warbler (*Acrocephalus schoenobaenus*) at Rybachy, Russia [48]

It has been assumed that nocturnal migrants depart soon after sunset (e.g. [4, 10]). During evening twilight a maximum number of navigational cues become available. Then birds are able to recalibrate their different compass systems, e.g., geomagnetic and celestial compass [27, 28], using the polarization pattern of visible light during twilight [29, 30]. Thus, one would expect a massive exodus of nocturnal migrants in the first two hours of the night as indeed commonly observed in radar studies [9, 31–35]. However, using ground surveillance radar the departure behavior of a priori selected individuals cannot be monitored, and the birds’ actual departure from the ground is difficult to detect [36]. Further, the vertical speed of individual birds – providing information about ascending or descending tendencies – is generally balanced after the initial exodus [15]. Thus, quantifying the proportion of individuals starting their nocturnal migratory flights after the exodus by radar is not straightforward and likely to be underestimated in the course of the night. The temporal distribution of nocturnal departures recorded by visual observations during the night, high mist-nets and radio tracking indicates that the timing of nocturnal departures is not exclusively confined to the specific period of 1 to 2 h after sunset [37–47], but see [48] (Fig. 1). Although most birds directly observed departing or caught in high mist-nets might have intended to depart from the study site, other nocturnal behaviors cannot be excluded. Recently, several types of nocturnal behavior, apart from real departures in seasonally appropriate directions, have been described including reverse movements, nocturnal explorative flights and landscape-scale stopover movements [43, 49, 50]. Such uncertainties can be minimized by radio-tracking individual birds provided that nocturnal movements on the landscape-scale are not misinterpreted as actual departures for migratory flight [49, 50].

The observed variation in nocturnal departure time raises the question of which proximate causes help to explain the pattern (Fig. 1). Since most nocturnal songbird migrants travel between their breeding areas and wintering grounds without any parental or social guidance, they rely entirely on their innate migration program to reach their migratory goal, at least during their first autumn migration [51]. The innate migration program of these birds includes inherited dispositions for migratory directions, duration of migration, and migratory fueling, which are governed by endogenous rhythms (for reviews see: [51–57]). Further, the innate migration program determines how birds react jointly to different intrinsic factors and the currently encountered environment in terms of migratory direction [42, 58, 59], duration of migration, and stopover decisions [60–62], reviewed in [57, 63]. The combination of innate rhythms and environmental conditions predetermines the day-to-day departure decisions in migrants along their route and over the season (e.g. [63–68]). However, little is known about the cues and underlying mechanisms that shape the variation in nocturnal departure time, i.e. departure decisions on the within-night level.

**Fig. 2:**
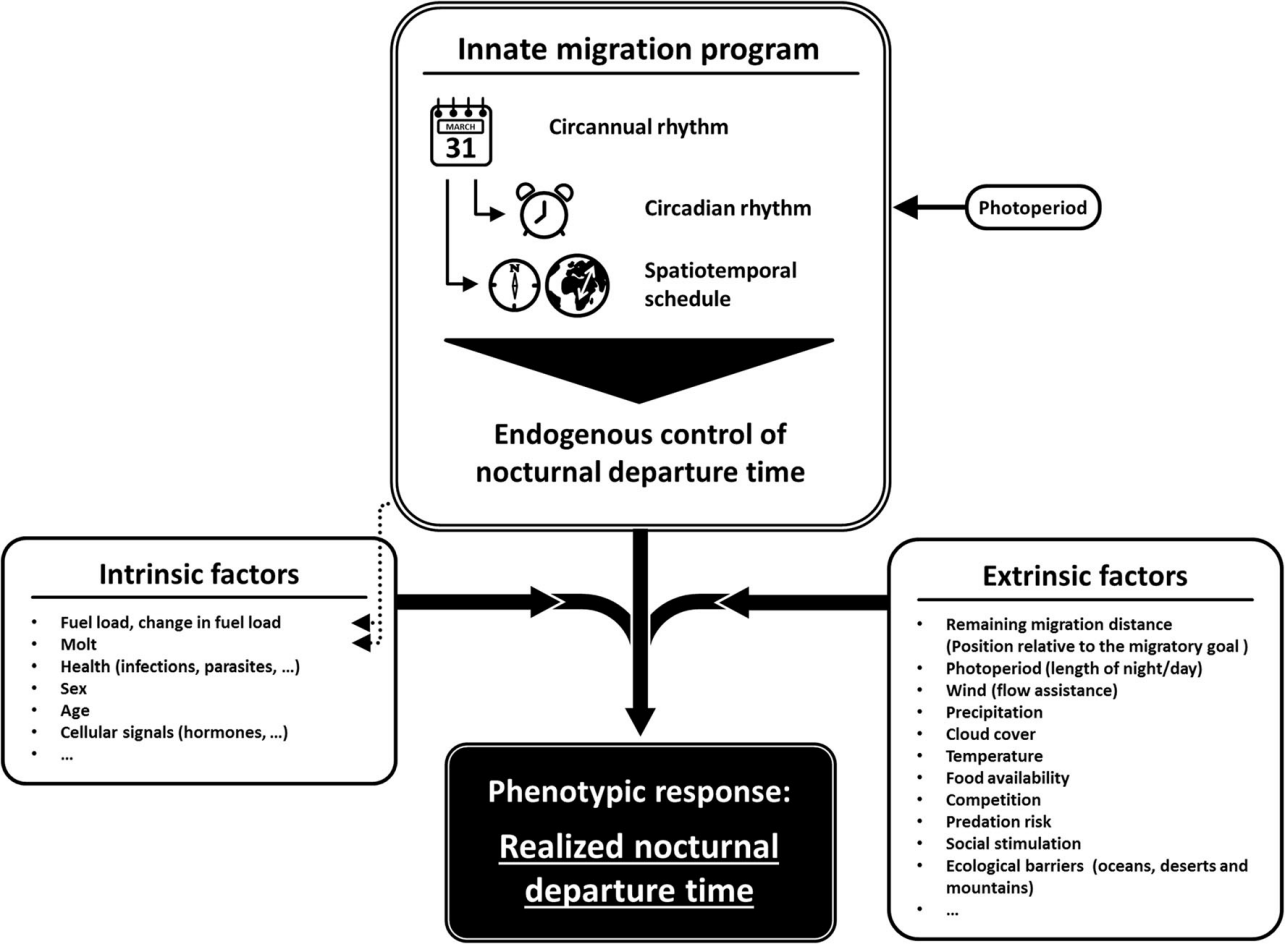
Schematic conceptual framework for the regulation of nocturnal departure time in songbird migrants. The innate program provides the circannual, circadian rhythms and spatiotemporal schedule of migration. The photoperiod is used to calibrate or reset the innate migration program. The seasonal variation in birds’ fuel load and the seasonal-specific sequence of molt are regulated among other traits by the innate rhythms (*broken arrows*). The realized nocturnal departure time represents the interplay of both the intrinsic and extrinsic factors modulating the endogenous stimuli

Here, we aim to review the current knowledge about variation in nocturnal departure time to provide a first conceptual framework of how this trait may be regulated in migratory songbirds (Fig. 2). We postulate that once a bird has decided to resume migration, the nocturnal departure time is endogenously controlled by the underlying innate rhythms of nocturnal activity. We additionally expect that a set of intrinsic and extrinsic factors modify this endogenously controlled basic schedule of nocturnal departure time [42, 45]. The result would be the realized nocturnal departure time, representing the phenotypic response of the individual bird to the experienced conditions (Fig. 2). With this work we summarize previous findings and present a theoretical basis for future investigations on proximate causes and underlying mechanisms of variation in the nocturnal departure time of migratory songbirds.

## Endogenous control of nocturnal departure time

The innate migration program of birds comprises both circannual (yearly) and circadian (daily) rhythms acting as biological clocks that provide the underlying temporal basis for migration [54]. The circannual rhythm times the different life history stages (e.g. breeding, molt, migration, wintering) of the annual cycle, including the initiation of migration and the physiological adaptations for migratory fueling (reviewed by [69]). In nocturnal migrants that are usually day-active outside of their migration period (but see [70]) the circannual rhythm is assumed to cause changes in the circadian rhythm which involve the development of nocturnal activity for migration [54]. Both rhythms can run independently of any environmental cues as shown in long-term common-garden experiments (e.g. [54, 71, 72]). However, without any external cues both rhythms fail to exactly track the natural migratory schedule after some time [54].

To investigate the innate migration program and its temporal rhythms, most studies made use of the fact that caged birds in migratory disposition, i.e., during the natural migration periods, show spontaneous seasonal variation in body mass [71, 73] and restless movements consisting of wing whirring and hopping during the night, when they would usually migrate [74, 75]. This nocturnal migratory restlessness (“*Zugunruhe*”) was shown to be a good general proxy for the migratory behavior that a bird would show in the wild. In naïve birds on their first autumn migration, intensity and duration of nocturnal migratory restlessness throughout the season reflect the inherited migratory distance [71, 72, 76]. In wild songbirds caught on migration, the intensity of nocturnal migratory restlessness predicts the subsequent departure likelihood from a stopover site [77]. Furthermore, the start of nocturnal migratory restlessness of caged songbirds caught on migration is positively correlated with their actual departure time in the following night [78]. Analyzing patterns of nocturnal migratory restlessness therefore represents the key method for studying endogenously controlled migratory behavior in birds, including its variation among different populations and individuals [57].

As the spatiotemporal organization of migration is predetermined by the innate migration program [54, 57], we postulate that the timing of nocturnal departure is likely endogenously controlled as well (Fig. 2). However, so far we lack systematic experiments focusing on this trait in naïve birds. The diel activity patterns of Garden Warblers (*Sylvia borin*), a long-distance songbird migrant, kept under a naturally changing day-light regime in autumn may suggest an endogenously controlled advancement in the start of nocturnal migratory restlessness with the progress of the migration season [54]. It needs to be considered that the corresponding data were not analyzed in this respect and sample size was low. In the European Quail (*Coturnix coturnix coturnix*), a nocturnal long-distance migrant among landfowl, individuals from a captive stock that experienced natural day length during spring were consistent in their start of nocturnal migratory restlessness over at least six consecutive nights [79]. A similar consistent pattern was found in a nocturnal songbird migrant, the Common Redstart (*Phoenicurus phoenicurus*) [80]. Individuals were caught on migration and subsequently kept under constant dim light conditions without access to environmental cues for three consecutive day-and-night cycles. Their nocturnal migratory restlessness consistently started within a narrow time window over the entire trial [80]. This suggests that birds may use their circadian clock to time the initiation of migration within the night. Whether this observed within-individual consistency in the start of nocturnal migratory restlessness is also reflected in the realized nocturnal departure time under free-flying conditions remains unknown. So far, there is only anecdotal evidence from a single Swainson’s Thrush (*Catharus ustulata*) being radio-tracked for over 1,500 km during seven consecutive nights [81]. On six of the seven nights, nocturnal departure times ranged over a narrow time window, from nine to 13 min after evening civil twilight, i.e, when the sun was at least 6° below the horizon [81].

The basic endogenous schedule of nocturnal departure time is likely to be affected by the birds’ migratory strategy (e.g. short versus long-distance migrants) and their total migration distance. The total migration distance can be regarded as a species- or population-specific trait of the innate migration program, because both the amount and duration of nocturnal migratory restlessness represent the population-specific migration distance [71, 72, 76]. Furthermore, there is a population-specific increase in total speed of migration with total migration distance [16, 82–84]. This may suggest that not only the amount and duration of nocturnal migratory restlessness, but also the travel speed, may be endogenously controlled in relation to a bird’s total migration distance. As travel speed is a function of nocturnal departure time (see above), the mechanism driving the population-specific speed of migration might be regulated by a population-specific nocturnal departure time (cf. [44]), if landing times do not vary to the same extent. This is, however, still completely unknown. Correlative support for a population-specific nocturnal departure time has been found in the Northern Wheatear (*Oenanthe oenanthe*), a long-distance nocturnal songbird migrant of which all populations winter in sub-Sahelian Africa. A comparison between Northern Wheatears breeding in Alaska (USA), northeastern Canada and Europe revealed that there is a positive association between total speed of migration (ranging between 115–200 km/day) and the respective total migration distance (ranging between ca. 4000 – 14500 km) [16, 82–85]. Furthermore, the nocturnal departure time advanced towards sunset with an increase in the population-specific total migration distance and the corresponding total speed of migration of the different wheatear populations [44]. Hence, species/populations with a higher speed of migration, related to a longer total migration distance, are likely to depart earlier and/or with less temporal variation in the beginning of the night [44]. Visual observations of departures from the Courish spit (Kaliningrad region, Russia) during five autumn migration seasons support this pattern, as long-distance migrants took off earlier and with less temporal scatter within the night than short-distance migrants [86]. Causal investigations are required to systematically test whether variation in nocturnal departure time can be indeed attributed to total migration distance.

## Phenotypic response to intrinsic and extrinsic factors: the realized nocturnal departure time

**Fig. 3:**
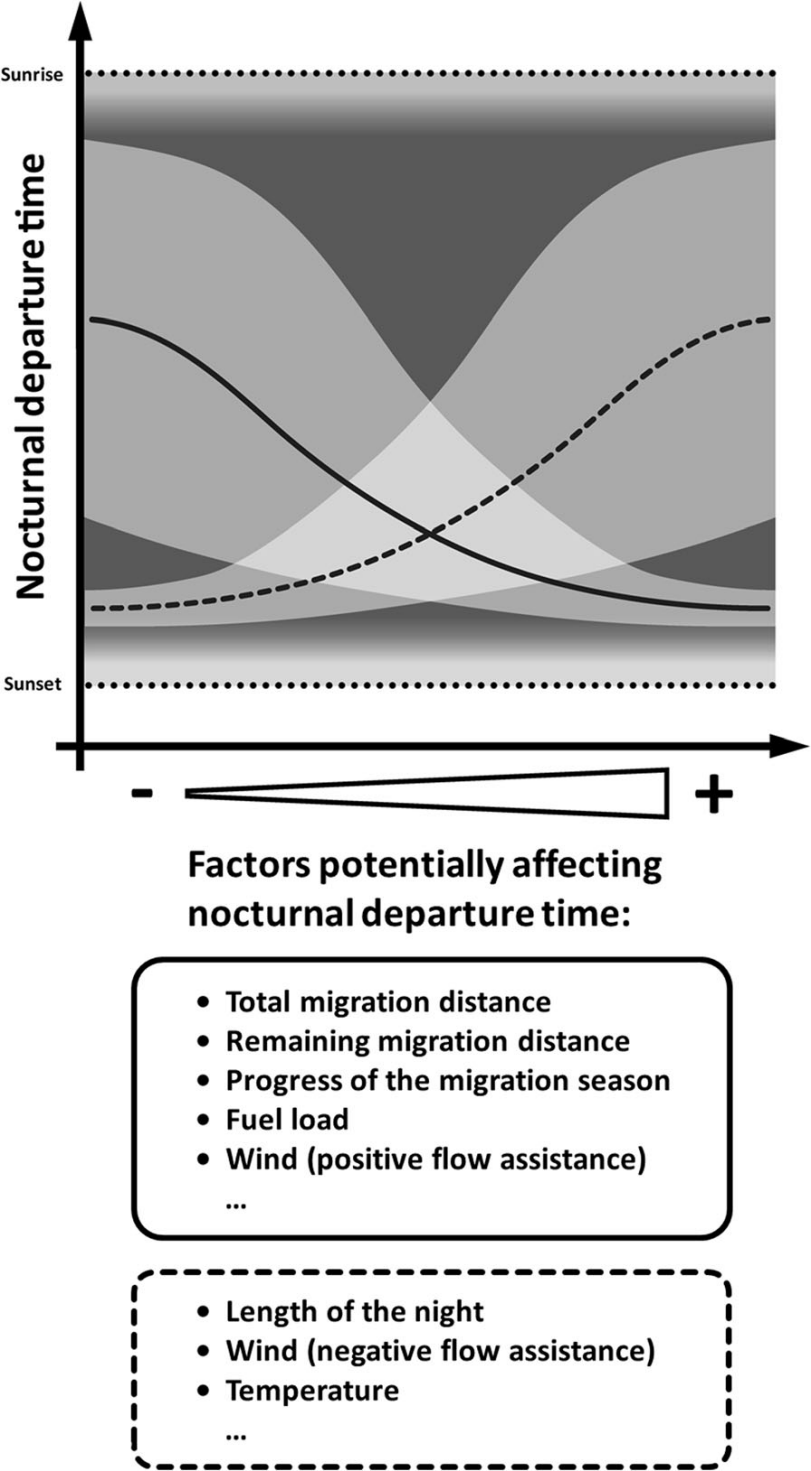
Predictions about the potential effect of different intrinsic and extrinsic factors on nocturnal departure time of songbird migrants. Predicted effects on mean nocturnal departure time (*solid and dashed line*) and its variation (*shaded light gray*) refer to factors in the respective boxes (*solid and dashed fringe*)

During migration, many birds encounter a variety of habitats that differ from their natal areas [87–89]. This environmental variation may affect a bird’s overall body condition and stopover decisions [68, 89]. The between-individual variation in nocturnal departure time observed under the same set of intrinsic and extrinsic factors is likely explained by the individual differences in their innate migration program yielding individually different phenotypic responses. Between-individual differences in the intrinsic and extrinsic factors additionally contribute to the observed variation in nocturnal departure times [39, 42, 44–47] (Fig. 3). Here we review results on the intrinsic and extrinsic factors that are known to influence nocturnal departure time in birds. In addition, the factors that have been shown to affect departure decisions on the day-to-day level [63] are discussed with respect to their potential effect on the nocturnal departure time. We also consider cage studies of individual variation in both the amount and the start of nocturnal migratory restlessness.

### Intrinsic factors

#### Fuel load

In addition to its effects on decisions on the day-to-day level [63, 90], fuel load also influences within-night departure decisions. Individuals with a high fuel load depart earlier within the night and with less temporal variation than individuals with a lower fuel load [42, 45, 46], but see [39–41]. Birds with high fuel loads, enabling a night-long flight towards their migratory goal, are thought to use the entire night for flying. They likely decide within a short time period after sunset to resume migration [42]. In contrast, birds with low fuel loads – having still decided to leave the current stopover site – could depart any time of night, possibly travelling shorter distances to nearby stopover sites that are potentially more favorable for refueling [49, 50]. We argue that “lean” birds continuously decide when to leave the current site during the course of a night. Such behavior would explain the high variation in their nocturnal departure time. For these birds a late departure may be advantageous, as it could ensure arrival at the next stopover site with some safety margin of fuel [41, 48]. Further, decisions for late departures may be driven by the advantage of early morning arrivals that allow visual selection of suitable stopover sites. Both would help to minimize the risk of starvation during migration.

Most published studies that focus on the effect of fuel load on nocturnal departure times are based on between-individual (cross-sectional) correlations [42, 45–47]. Since the individual change in fuel load over a bird’s stopover duration is positively correlated with the concurrent change in nocturnal migratory restlessness [91], and since an individual increase in restlessness is equivalent to an individually higher departure probability [77], we stress that the within-individual variation in fuel load is one important factor predicting individual nocturnal departure time. However, detecting within-individual variation in fuel load and nocturnal departure time is notoriously difficult in field studies. Avoiding these difficulties by temporarily caging migrating Northern Wheatears demonstrated a strong negative within-individual effect of fuel stores on the start of migratory restlessness, indicating that an increase in individual fuel stores induced an advanced start of nocturnal migratory restlessness on the level of the individual [92]. As the latter is a reliable approximation for the nocturnal departure time [78], the individual change in fuel load is likely an important intrinsic factor shaping individual variation in nocturnal departure time (Fig. 3). Information about the current fuel load and its changes during a bird’s stopover will be incorporated in the expression of innate migratory rhythms (Fig. 2) and the resulting behavioral response, i.e., departure decisions on the day-to-day level and during the night.

#### Molt, health, sex, and age

There are other intrinsic factors, like molt, health, sex, and age, influencing departure decisions on the day-to-day level and the general movement ecology of migrants (e.g. [63, 93–95]). Although these have not been related to nocturnal departure time, we briefly discuss their potential influence here.

Molt is an energy and time demanding process in the annual cycle of birds [96]. As molt also compromises the flight ability [97–99] and likely increases predation risk [100], most birds molt in the breeding areas and/or on the wintering grounds prior to migration or suspend the molt during migration [101–105]. Migrating during molt or with suspended molt increases flight costs [97–99] and thereby decreases travel speed. Birds either set off early in the night to compensate for the low travel speed, or they time their nocturnal departure irrespective of molt status but reduce the intended travel distance per night. Thus, the potential effect of active or suspended molt on the variation of nocturnal departure time remains ambiguous.

Infections, diseases, and parasites can seriously affect the health of birds, and consequently their migratory performance [106]. A poor overall body condition as a result of a poor health status likely increases the variation in the timing of nocturnal departure, because sick birds may have to allocate energy for stimulating their immune system [106, 107] at the cost of fuel deposition for migratory flights.

Sex is an important intrinsic factor influencing the phenology of most migratory birds [108–110]. In spring, males typically arrive at the breeding area and at stopovers earlier than females [26, 85, 111–114]. In addition to the sex-specific initiation of spring migration at the wintering grounds, there is evidence for a higher speed of migration in males than in females [85]. Higher migration speed in males may be realized either by shorter stopover durations, increased airspeed or more time spent flying during each migratory flight bout. The latter can be accomplished by an earlier or less variable nocturnal departure time in males. If so, we predict that the sex-specific differences in nocturnal departure time may be related to sex-specific differences in their travel speed. Yet, we lack any evidence for such a sex-specific difference in nocturnal departure time.

The age of a migratory bird affects the initiation of migration [115], the selection of the route [64, 115–117], and the selectivity with regard to favorable wind conditions for departure [93]. In general, young individuals show more variation in migratory traits than old birds [93, 116, 117], although there is no clear pattern in respect of the nocturnal departure time [45]. However, potential age effects are difficult to assess, since they can be attributed either to learning [116, 117] or to selective disappearance reducing genetic and phenotypic variation in older age classes [118]. Longitudinal data, i.e., tracking the time when individuals resume migration within the night over many years, are required to correctly assess the effect of age on nocturnal departure time.

### Extrinsic factors

#### Remaining migration distance and progress of the season

The remaining migration distance to the seasonally appropriate migratory goal is subject to a continuous change along the migration route. A migrant’s general stopover ecology is modulated by the individual’s position [119–121] and the time within the season [64]. This demonstrates the capability of birds to estimate their relative whereabouts along the migration route (reviewed in [122]). Simulating a geomagnetic displacement in a “common-garden experiment” showed that Northern Wheatears adjusted their amount of nocturnal migratory restlessness in relation to the simulated remaining migration distance [121]. Whether the nocturnal departure time is also affected by the remaining migration distance remains unknown. In spring, individuals that have a long remaining migration distance and/or that are late in the migratory season are thought to increase their fitness by minimizing the remaining time required to reach the breeding area still within the reproductive time window [110]. By departing closer to sunset, individuals can maximize their potential nocturnal flight duration and thereby advance their arrival in the breeding area. Given this, we predict that birds with longer remaining migration distances should depart earlier in the night and/or show less temporal variation than those with shorter remaining migration distances (Fig. 3). Furthermore, individuals migrating late in the season may also show earlier nocturnal departure times so as to increase their migration speed relative to individuals of the same population migrating early in the season (Fig. 3).

#### Length of night

For nocturnal migrants, the length of the night (being inextricably linked with time within season) generally predetermines the potential maximum flight duration for a migratory flight bout, although birds may prolong their flights into the day when crossing ecological barriers [9, 15, 17–20]. It has therefore been hypothesized that with a decrease in night length, nocturnal departure time is more concentrated shortly after sunset [37, 39] (Fig. 3). As a consequence, the predicted patterns are opposite for spring and autumn (Fig. 3). A telemetry study with European Robins passing the Courish spit (Kaliningrad region, Russia) revealed a seasonal difference in the median nocturnal departure time which was related to seasonal differences in night length [39]. However, environmental conditions [123] and the main endogenous drivers for an early nocturnal departure likely also differ between seasons (cf. [71, 124]) and so may explain the observed seasonal differences.

#### Weather

Weather variables, such as wind, air pressure, cloud cover, precipitation, and air temperature, have been shown to strongly influence the departure behavior of birds [22, 63]. Favorable wind conditions increase the departure probability from a stopover site, whereas birds prolong their stopover under unfavorable wind conditions (e.g. [22, 50, 65, 125–129]). Likewise, correlative evidence suggests that birds advance their nocturnal departures towards sunset to maximize the potential flight duration under favorable and/or improving wind assistance [42, 130] (Fig. 3). Wind is the result of differences in air pressure. Therefore, a change in air pressure is usually predictive of the upcoming wind conditions. Birds are able to detect changes in air pressure [131, 132]. Migrants may use this cue for their departure decisions [22, 133, 134]. Based on a study with European Robins it has been suggested that a change in air pressure may also affect the exact timing of nocturnal departures [41]. Birds experiencing a drop in air pressure during the day were found to set off late at night [41]. In general, it seems that favorable winds and rising air pressure promote early nocturnal departure times. However, the effect of deteriorating wind conditions (i.e., headwinds) on nocturnal departure time is difficult to predict and likely depends on the magnitude and direction of the alternation.

The effect of cloud cover on nocturnal departure time has mainly been considered in relation to the visibility of celestial cues [37, 39–41]. Species that use a celestial compass (e.g. [135, 136]) may delay their nocturnal departure under an overcast sky until stars become visible. Such a behavioral response was observed in European Reed Warblers (*Acrocephalus scirpaceus*) [37], but not in European Robins [39–41]. It remains therefore unclear whether or not cloud cover itself has a significant impact on nocturnal departure time. Alternatively, the probability of precipitation related to the magnitude of cloud cover may affect the birds’ departure decision [127, 137], because rain represents a severe hazard during flight [138]. Yet, no study could demonstrate that a high probability of precipitation affects the nocturnal departure time of birds.

With a drop in air temperature both the day-to-day departure probability from stopover sites [44, 83] and the amount of nocturnal migratory restlessness [139, 140], indicative for the departure probability of birds [77], have been shown to increase. Such a reaction is regarded as an energy-saving strategy to minimize the costs of thermoregulation [141], as the energy expenditure of birds decreases with increasing temperature [141, 142]. For the same reasons we hypothesize that birds time their nocturnal departure closer to sunset when experiencing cold conditions on the ground for the same reasons as outlined above (Fig. 3). The effect may be more pronounced during the autumn migration season, when warmer conditions are expected towards the seasonally appropriate migratory direction, than in spring.

#### Food availability, competition, and predation risk

Other factors such as food availability, inter-/intra-specific competition and predation risk have been shown to influence a bird’s day-to-day departure decisions [63, 143]. These three factors characterize the quality of the current stopover site which in turn affects a bird’s fueling rate and the resulting departure fuel load (e.g. [144–146]). Hence, we assume that they may act on the nocturnal departure time via their effect on the bird’s fuel load.

#### Social stimulation

Many bird species utter specific calls during flight, especially during the migratory periods. These flight calls are assumed to help birds keeping contact in loose flocks during nocturnal migration [147]. Furthermore, these calls seem to stimulate migratory behavior of conspecifics, as indicated by a study on captive Bobolinks (*Dolichonyx oryzivorus*) [148]. In this study, birds responded to flight calls of conspecifics by an immediate increase in their nocturnal activity in the cage, if they had shown nocturnal migratory restlessness before. If they had been inactive during the previous night, flight calls induced only a weak activity response [148]. This might indicate that migratory songbirds, which are ready for a nocturnal flight bout, can get stimulated to depart by social stimuli like the flight calls of their conspecifics. Whether flight calls affect the actual departure times of migrants at night remains ambiguous.

#### Ecological barriers

On their migratory journeys, most long-distance songbird migrants will have to negotiate species-specific adverse habitats for resting and refueling to reach their respective breeding areas or wintering grounds. At the extreme, these habitats may offer limited or even no opportunity for landing and feeding, as do large water bodies (oceans or large lakes) and deserts for migratory land birds. The crossing of such ecological barriers requires sufficient fuel and the ability to select for favorable weather conditions [42, 63, 71, 128, 129, 137, 149, 150]. Hence, migrants that encounter an ecological barrier need to jointly integrate intrinsic (fuel load) and extrinsic factors (wind, precipitation, air pressure) for their departure decision [42], as a mistake may have lethal consequences for the individual. If birds carry insufficient fuel and/or encounter unfavorable weather conditions for such a crossing, they may either prolong their stopover in wait for better conditions [66], perform reverse migration in search of a more suitable stopover site [42, 45, 151–155] or circumnavigate the barrier by a detour [156]. These behaviors are usually observed in rather lean individuals and the timing of departure ranges over the whole night [42, 45, 47]. In contrast, migrants with sufficient fuel loads for the crossing and experiencing favorable weather time their departures early within the night with only little temporal variation [42, 45, 46]. In doing so, these birds maximize the night time available for flying across the barrier and thereby minimize their exposure to the disadvantages of daytime flights, i.e., more turbulent air [10] and higher predation risk [13].

## Conclusions

Our concept suggests that the basic schedule of nocturnal departures is regulated by the circannual and circadian rhythms of the innate migration program. The sum of intrinsic factors cumulatively describes a bird’s current overall body condition, i.e., its general readiness for a migratory flight. This readiness together with the environmental conditions described by the extrinsic factors is likely fed back to the innate migration program and thus, both modulate jointly the endogenously controlled nocturnal departure time (Fig. 2). The observed realized nocturnal departure time of an individual bird is the result of this process.

Variation in nocturnal departure time is likely explained by individual differences in the innate migration program and the individually different phenotypic reaction norms to both the intrinsic and extrinsic factors. At present, it seems that the individual change in fuel load and the individually experienced wind conditions are the main drivers for the variation in nocturnal departure time. Potential effects of other weather parameters are difficult to assess, as they are all highly correlated with each other. Thus, there is a severe statistical issue, in terms of multicollinearity [157]. There is further a biological issue, as it remains difficult to distinguish whether individual birds respond to one or more weather parameters at the same time in correlative studies [89].

Given that the start of nocturnal migratory restlessness is positively correlated with the nocturnal departure time, allows the identification of factors causing individual variation in the former trait and transferring these results to nocturnal departure times [78]. This provides the potential to investigate causal relationships and easily separate between within- and between-individual variations in future experimental studies [92].

Under free-flying conditions, nocturnal movements not related to departures for migratory flights add an unknown amount of variation to our estimates of the nocturnal departure time. Ideally, these can be disregarded. Stopover sites in front of an ecological barrier allow distinguishing between departures for migratory flights and non-migratory movements. However, the crossing of an ecological barrier constitutes a special case, as the barrier itself is an environmental factor of major significance. Results from these sites may not be directly transferable to the nocturnal departure behavior of birds migrating across benign landscapes. New technical developments for tracking small songbirds on a broad landscape scale [158] or even globally [3, 159] will help to disentangle the different types of migratory and non-migratory nocturnal movements. This will significantly improve our understanding of the timing of nocturnal departures.

Technical improvements and miniaturization of accelerometers implemented in small tracking devices [160] will further enable studying individual departure and landing times in migratory birds simultaneously. The resulting actual migratory flight durations and the corresponding distances covered will detail the between and within-individual variation in migration strategies, which may extend our understanding of the flexibility of migratory behavior substantially.
